# The Clinical Significance of HLA Compatibility Scores in Lung Transplantation

**DOI:** 10.3389/ti.2024.13484

**Published:** 2025-01-03

**Authors:** Liesbeth Daniëls, Hanne Beeckmans, Andrea Zajacova, Pieterjan Kerckhof, Saskia Bos, Maarten Naesens, Bart Vanaudenaerde, Frans Claas, Robin Vos

**Affiliations:** ^1^ Histocompatibility Laboratory (HLA), Clinical Biology, CHU UCL Namur Site Godinne, Namur, Belgium; ^2^ Faculty of Medicine and Health Sciences, University of Antwerp, Antwerp, Belgium; ^3^ Department of Chronic Diseases, Metabolism and Ageing, Laboratory for Respiratory Diseases and Thoracic Surgery, Faculty of Medicine, KU Leuven, Leuven, Belgium; ^4^ Department of Respiratory Diseases, University Hospitals Leuven, Leuven, Brussels, Belgium; ^5^ Department of Microbiology, Immunology and Transplantation, Faculty of Medicine, KU Leuven, Leuven, Belgium; ^6^ Eurotransplant and Eurotransplant Reference Laboratory, Department of Immunology, Leiden University Medical Center (LUMC), Leiden, Netherlands; ^7^ Department of Translational Research in Immunology and Inflammation, University of Antwerp, Antwerp, Belgium

**Keywords:** lung transplantation, HLAMatchmaker, HLA-EMMA, PIRCHE-II, KIR ligand calculator

## Abstract

Lung transplantation is a life-saving therapeutic option for many chronic end-stage pulmonary diseases, but long-term survival may be limited by rejection of the transplanted organ. Since HLA disparity between donor and recipient plays a major role in rejection, we performed a single center, retrospective observational cohort analysis in our lung transplant cohort (n = 128) in which we calculated HLA compatibility scores for B-cell epitopes (HLAMatchmaker, HLA-EMMA), T-cell epitopes (PIRCHE-II) and missing self-induced NK cell activation (KIR Ligand Calculator). Adjusted Cox proportional hazards model was used to investigate the association between mismatched scores and time to development of donor-specific antibodies (DSA) post-transplant, time to first biopsy-proven acute rejection episode, freedom from CLAD, graft survival and overall survival. For time to first DSA, HLA-EMMA DQB1 scores and PIRCHE-II DQB1 scores were significantly associated with more rapidly developing anti-HLA-DQ antibodies. HLA-EMMA DQB1 score was significantly associated with worse survival. KIR ligand Host-versus-Graft (HvG) mismatches was significantly associated with worse graft survival (CLAD or death) and shorter time to first biopsy-proven rejection when 2 mismatches were present. We demonstrated that HLA-DQB1 compatibility scores and KIR ligand HvG 2 mismatches may allow for identification of recipients at risk of poor long-term outcomes after lung transplantation.

## Graphical Abstract

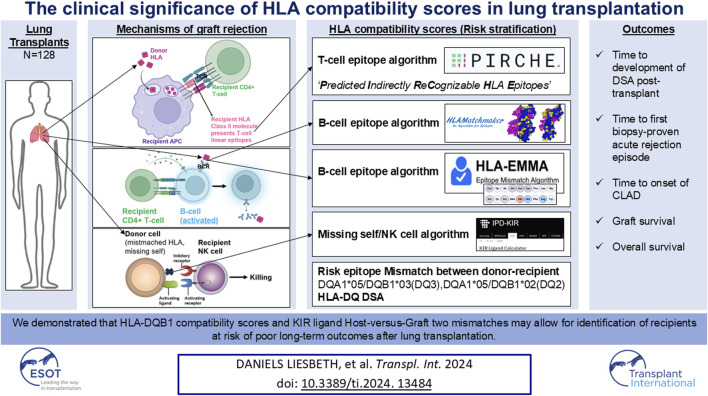

## Introduction

Lung transplantation is a life-saving therapeutic option for many chronic end-stage pulmonary diseases. However, long-term survival after lung transplantation is the worst of all solid organ transplantations and is, in large part, limited by chronic rejection, or so-called chronic lung allograft dysfunction (CLAD) [1]. CLAD encompasses a range of pathologies causing a transplanted lung allograft to not achieve or maintain its normal function, which clinically manifests as airflow obstruction and/or restriction [2].

Human leukocyte antigen (HLA) disparity between donor and recipient affects the alloimmune response and consequently has an impact on graft outcome [3]. The foreign HLA antigens of the donor are recognized by the adaptive immune system of the recipient, which - when activated - can lead to organ injury by rejection; and finally, the failure of the transplanted organ [4]. Immunogenicity is the ability to induce an antibody response while antigenicity is based on the actual interaction between antibody and an antigen and varies according to the recipient’s self HLA and the mismatched donor HLA [5]. The portion of the HLA molecule that interacts with anti-HLA antibodies, the binding site, is called “epitope.” An “eplet” represents the smallest functional unit contributing to the antibody specificity and forms a smaller portion ( ∼ 3 Å diameter) of the larger overall epitope ( ∼ 15 Å diameter) [6].

Besides B cell epitopes, T cell epitopes may also play a role in antibody formation, since donor-specific anti-HLA antibodies (DSA) production occurs via the indirect allorecognition pathway in which foreign HLA is processed by the recipient’s antigen-presenting cells and presented by HLA class II to CD4^+^ T cells, followed by B cell activation, plasma cell formation and antibody production. As such, HLA-derived T cell epitopes, designated as PIRCHE-II (Predicted Indirectly ReCognizable HLA epitopes presented by HLA class II molecules), also play a role in generation of *de novo* (dn)DSA and graft failure [7–9]. Circulating DSA bind to allogeneic HLA on donor cells’ surface (e.g., endothelial cells), inducing endothelial cell activation, and subsequent recruitment of innate immune cells and complement factors. Next, recruited innate immune cells bind to the HLA-DSA and release cytotoxic granules (a process called antibody-dependent cell-mediated cytotoxicity/ADCC), and/or complement fixation and activation occurs, leading to formation of a membrane attack complex (a process called complement-dependent cytotoxicity/CDC). Both these pathways in the process of antibody-mediated rejection (AMR) result in cytolysis (cell death) of the targeted “non-self” cells. Moreover, T cells within the draining pulmonary lymph nodes are also activated after binding with membrane-bound allogeneic HLA on antigen-presenting cells, either donor- or recipient-derived, that have migrated from the lung allograft. Activated T cells then enter the blood circulation and may infiltrate the allograft inducing a local inflammatory response termed acute (T cell-mediated) cellular rejection (ACR).

In addition to antibody-mediated and T cell-mediated rejection, as described above, Koenig et al. [4] demonstrated in kidney transplants that missing self-induced natural killer (NK) cell activation promotes the development of graft microvascular inflammation that has exactly the same harmful impact on organ survival as non-complement activating anti-HLA DSA, the principal cause of late transplant loss. In steady state, the interaction of inhibitory Killer-cell immunoglobulin-like receptors (KIRs) with self-HLA class I molecules of surrounding healthy cells provides a negative signal. On the contrary, the downregulated expression of HLA class I molecules associated with tumoral transformation or viral infection triggers NK cell activation, which results in destruction of the target cell, a process called response to ‘missing self’. In clinical transplantation, however, graft endothelial cells are unable to deliver inhibitory signals to recipient NK cells because of different (mismatched) HLA class I molecules. This imitates the ‘missing self’ for NK cells.

We assume that primed NK cells in the lung transplant recipient’s circulation (due to ischemia/reperfusion injuries and/or prior (viral) infections) may also promote endothelial damage in lung allografts, and that “missing self” thus should also be considered as a risk factor in the process of rejection after lung transplantation. Patients with missing self-induced rejection will not respond to the costly and tedious treatment of AMR [4]. Missing self-induced NK cell activation is mTORC1- dependent, and mTOR inhibitors may prevent development of this type of chronic vascular rejection [4]. Therefore, it is critically important to clinically identify this process in lung transplant patients at risk for/with rejection, to accordingly adjust treatment (i.e., pathway-directed therapy) in these patients.

Since HLA disparity between donor and recipient plays a major role in rejection, as evidenced by complement activating anti-HLA antibodies (CDC), ADCC caused by anti-HLA DSA, T cell-mediated cellular rejection and missing self-induced rejection by NK cells, it is important to explore which HLA software tools can be used to calculate HLA compatibility scores, in order to identify high-risk patients, fine-tune each patient’s immunosuppressive regimen (personalized treatment) and further improve lung transplantation outcomes [10].

As data regarding HLA software-based risk identification are scarce in lung transplantation, we performed a single center, retrospective observational cohort analysis in our lung transplant cohort.

## Materials and Methods

### Cohort

All consecutive adult lung transplant recipients at the University Hospitals Leuven between 1 January 2015 and 31 December 2021 with written informed consent, clinical/histopathological data and donor/recipient DNA samples available for high-resolution HLA typing, were eligible for this observational cohort study. Recipients of combined transplantation (i.e., heart-lung, lung-liver, lung-kidney transplant) or lung transplantation after another transplantation were excluded. Following induction treatment with rabbit anti-thymocyte globulin, baseline immunosuppression consisted of a standard triple regimen consisting of tacrolimus, mycophenolic acid, and corticosteroids. No desensitization therapies for pretransplant anti-HLA antibodies were used. Patients at risk for cytomegalovirus (CMV) primo infection or reactivation (donor positive or recipient positive status) received prophylaxis with ganciclovir and valganciclovir for 3–6 months. During the first year post-transplant, all participants were followed clinically at monthly intervals and thereafter at three monthly intervals. Protocol-bronchoscopy with biopsies is routinely performed at 1, 3, 6, 12, 18, and 24 months, and in addition, indication-bronchoscopy with biopsies is performed upon clinical suspicion of graft rejection. Follow-up was censored at death or the censor date 31 December 2021. The study was approved by the Ethics Committee of the University Hospitals Leuven (BREATHE, KU Leuven) (S66760).

### HLA Typing

Until recently, high-resolution HLA typing was not routinely performed at the University Hospitals Leuven. Therefore, donor and recipient DNA samples obtained from blood were retrospectively genotyped at the EFI accredited HLA laboratory CHU UCL Namur Site Godinne using next-generation sequencing (GenDx NGSgo-MX11-3 on Illumina Miseq) for all loci (HLA-A, -B, -C, -DRB1, -DRB345, -DQB1, -DQA1, -DPB1, and DPA1). The HLA types of donor and recipient were reported as 2-field alleles for mismatch analysis, since it has been show that minor differences in one or more epitopes between donors and recipients at either locus are sufficient to generate an immune response [11].

### HLA Antibody Testing

HLA antibody results were retrospectively retrieved from the routine clinical database. Venous blood samples were collected routinely on day 0 and after transplantation on days 1–30–90–180–360–540–730, and annually thereafter as well as at intermediate time-points (i.e., when an indication-bronchoscopy with biopsies was performed or in case of suspected graft rejection). HLA antibody evaluation of all patient samples was performed with Immucor LIFECODES^®^ Lifescreen Deluxe kits. A positive screening for the presence of circulating HLA antibodies was followed by HLA antibody identification with Immucor LIFECODES^®^ LSA (Luminex Single Antigen) kits. All tests were performed and interpreted according to the manufacturer’s instructions. A Median Fluorescence Intensity (MFI) of ≥500 was used for assignment of HLA DSA positivity. All serum samples were treated with EDTA to eliminate the prozone effect.

### Bronchoscopic Surveillance

Patients underwent surveillance bronchoscopy with bronchoalveolar lavage and transbronchial biopsy as per our hospital protocol. ACR was diagnosed and graded according to the International Society for Heart and Lung Transplantation (ISHLT) Rejection Working Group with A- and B-grade component [12, 13]. Rejection of a severity of A1 or B1 or above was identified as ACR. AMR was diagnosed according to the 2016 ISHLT consensus [14] and include the presence of DSA and characteristic lung histology with or without evidence of complement 4d (C4d) within the graft. AMR was categorized into 3 mutually exclusive possibilities (definite, probable and possible). These categories were based on the degree of certainty related to the presence or absence of a number of pathologic, serologic, clinical and immunologic criteria (allograft dysfunction, other causes excluded, lung histology, lung biopsy C4d, DSA).

### HLA Compatibility Scores

For evaluation of the differential immunogenicity of HLA mismatches in lung transplantation we used the publicly available software tools based i.e., for B-cell epitopes “HLAMatchmaker v4.0 (HLA class I),” “HLAMatchmaker v3.1 (HLA class II)”^1^ [15] and “HLA-EMMA v1.06”^2^ [16], for T-cell epitopes “PIRCHE-II v3.3”^3^, and for missing self-induced NK cell activation [KIR ligand mismatch Host-versus-Graft (HvG)] “KIR Ligand Calculator” IPD-KIR Database (ebi.ac.uk) [17–19].

### Clinical Outcomes

The outcomes of interest we assessed were overall survival, time to onset of CLAD (freedom from CLAD), graft survival (defined as death or CLAD onset), time to development of dnDSA and time to biopsy-proven acute rejection (either cellular/ACR or antibody-mediated/AMR). CLAD was defined as a substantial and persistent decline in graft function (≥20%) in measured forced expiratory volume in 1 s value (FEV_1_) from the reference (baseline) value according to the latest ISHLT consensus [1]. Freedom from CLAD was calculated as the time between transplantation and the date of diagnosis of CLAD. Patients without CLAD were censored at the end of study follow-up or at the date of death. No CLAD patients included in our study underwent a retransplantation.

In a second part of the study, we investigated the detection of dnDSA occurrence post-transplant and the significance of specific HLA-DQ mismatches, since not all mismatches equally contribute to generation of donor-specific immune responses and mismatches of HLA-DQ likely exhibit the highest immunogenicity, specifically the DQA1*05/DQB1*02 and DQA1*05/DQB1*03 [20–22]. For this purpose, the University Hospitals Leuven clinical database was consulted retrospectively to evaluate whether and which HLA antibodies had been detected by Luminex technology, and risk-epitope mismatches (DQA1*05/DQB1*02 and DQA1*05/DQB1*03) were also evaluated in the current cohort.

During the analyses, known risk factors at transplantation, namely, pretransplant HLA sensitization, donor and recipient CMV status, recipient sex and age, were taken into account.

### Statistical Analysis

Patient statistics are presented as median and range or percentage, as appropriate. Cox proportional hazards model was used to investigate the association between mismatched scores and onset of first DSA post-transplant, time to first biopsy-proven acute rejection episode, survival and freedom from CLAD. Hazard ratios (HRs) (95% confidence interval (CI)) were used to define associations with scores and outcome variables of interest. Adjustment for known risk factors at transplantation were performed (sex, age, HLA sensitization and CMV status). In all models, a p-value of <0.05 was considered significant. RStudio version 4.3.1 was used for all statistical analyses and Kaplan-Meier survival curves.

## Results

### Cohort

The study cohort comprised 128 lung transplants with a median age of 59 (range 18–66) in whom pretransplant DSA were detectable in 7 cases (5%). Chronic obstructive pulmonary disease (emphysema) (63%) was the most common indication for lung transplantation. Nineteen percent of patients (n = 24) developed dnDSA post-transplant with anti-HLA-DQ as the predominant dnDSA (n = 20, 83%), after a median detection time of 271 days (range 10–1847). A total of 30 patients (23%) developed CLAD (n = 24 bronchiolitis obliterans syndrome, n = 5 restrictive allograft syndrome, n = 1 mixed). Patient cohort characteristics and parameters are summarised in Table 1.

**TABLE 1:** Patient characteristics (n = 128).

Parameter	Median (range or percentage)
Age at time of transplant, y (range)	59 (18–66)
Female sex, n (%)	67 (52%)
DSA positivity prior to transplant (HLA sensitization), n (%)	7 (5%)
Time between transplantation and death/end of study, y (range)	4.9 (0.4–7.0)
Time between transplantation and CLAD (n = 30), y (range)	3.9 (0.3–5.9)
De novo DSA positivity, n (%)	24 (19%)
HLA class I, n (%)	3 (13%)
HLA class II, n (%)	20 (83%)
HLA class I + II, n (%)	1 (4%)
HLA-DQ, n (%)	20 (83%)
**Subcohort without pre-transplant DSA (n = 121)**	
HLA antigen mismatches (A-B-DR), median (range)	5 (3–6)
HLA allele mismatches (A-B-C-DR-DQ-DP), median (range)	13 (6–17)
B-cell epitopes	
HLAMatchmaker total score, median (range)	24 (11–41)
HLAMatchmaker DQB1 score, median (range)	3 (0–9)
HLA-EMMA total score, median (range)	75 (23–131)
HLA-EMMA DQB1 score, median (range)	12 (0–32)
T-cell epitopes	
PIRCHE-II total score, median (range)	91 (32–189)
PIRCHE-II DQB1 score, median (range)	27 (0–82)
Missing self/NK cell	
KIR ligand HvG mismatch 1 MM, n (%) 2 MM, n (%)	65 (54%) 18 (15%)
Risk Epitope Mismatch (REM)	
DQA1*05/DQB1*03:01 (DQ7) MM, n (%)	31 (26%)
DQA1*05/DQB1*03 (DQ3)/DQB1*02 (DQ2) MM, n (%)	46 (38%)
DQA1*05/DQB1*03:01 (DQ7)/DQB1*02 (DQ2) MM, n (%)	47 (39%)

Legend: Data are presented as median and range or percentage, as appropriate. CLAD, chronic lung allograft dysfunction; DSA, donor-specific anti-HLA antibodies; HLA, Human Leukocyte Antigen; HvG, Host-versus-Graft; KIR, Killer-cell immunoglobulin-like receptors; MM, mismatch; PIRCHE-II, Predicted Indirectly ReCognizable HLA epitopes presented by HLA class II molecules; Y, years.

### HLA Compatibility Scores

Recipients without detectable pre-transplant DSA received a transplant with a median cumulative number of HLA-A, -B, -DR antigen mismatches of 5 (range 3–6) and HLA-A, -B, -DR, -DQ, -DP allele mismatches of 13 (range 6–17). HLAMatchmaker scores ranged from 11 to 41 with a median of 24, HLA-EMMA scores ranged from 23 to 131 with a median of 75, and PIRCHE-II scores ranged from 32 to 189 with a median of 91. Fifty-four percent of patients (n = 65) presented a KIR ligand mismatch in the Host-versus-Graft direction, of which 18 with 2 mismatches (15%).

Given the dominance of anti-HLA DQ antibodies in the *de novo* occurrence of HLA antibodies, we then focused on mismatches in the HLA-DQB1 locus. HLAMatchmaker scores ranged from 0 to 9 with a median of 3, HLA-EMMA scores ranged from 0 to 32 with a median of 12, and PIRCHE-II scores ranged from 0 to 82 with a median of 27.

### Association of HLA Compatibility Scores With Overall Survival, CLAD, and Graft Survival

Adjusted Cox proportional hazards models (adjusted for covariates sex, age, HLA sensitization and CMV status) regarding the outcomes of interest are summarized in Table 2.

**TABLE 2:** HLA compatibility scores and outcomes of interest.

Outcome	Covariates/HLA compatibility score	HR	95% CI	p
Overall survival				
	Age	1.08	0.70–1.68	0.7164
	Sex	0.63	0.23–1.71	0.3647
	CMV	1.19	0.33–4.27	0.7986
	HLAMatchmaker total score	1.07	0.57–2.01	0.8281
	HLAMatchmaker DQB1 score	1.70	0.87–3.31	0.1196
	HLA-EMMA total score	1.29	0.67–2.48	0.4461
	HLA-EMMA DQB1 score	2.49	1.11–5.59	0.0273
	PIRCHE-II total score	0.95	0.45–2.01	0.8842
	PIRCHE-II DQB1 score	1.88	0.90–3.90	0.0920
	KIR ligand HvG mismatch 1 MM 2 MM	2.02 2.79	0.69–5.91 0.95–8.17	0.1985 0.0616
	DSA anti-HLA-DQB1	1.90	0.60–6.00	0.2729
	DQA1*05/DQB1*03:01 (DQ7) MM	0.75	0.21–2.67	0.6521
	DQA1*05/DQB1*03:01 (DQ7)/DQB1*02 (DQ2) MM	0.61	0.19–0.93	0.4007
	DQA1*05/DQB1*03 (DQ3)/DQB1*02 (DQ2) MM	0.59	0.19–1.86	0.3673
CLAD				
	Age	1.28	0.87–1.87	0.2112
	Sex	0.74	0.35–1.56	0.4292
	CMV	1.20	0.48–2.98	0.6979
	HLAMatchmaker total score	1.00	0.61–1.65	0.9979
	HLAMatchmaker DQB1 score	0.74	0.43–1.28	0.2856
	HLA-EMMA total score	1.05	0.63–1.76	0.8571
	HLA-EMMA DQB1 score	0.77	0.41–1.45	0.4200
	PIRCHE-II total score	1.03	0.59–1.78	0.9199
	PIRCHE-II DQB1 score	0.97	0.54–1.73	0.9228
	KIR ligand HvG mismatch 1 MM 2 MM	1.03 2.16	0.49–2.19 0.91–5.10	0.9323 0.0799
	DSA anti-HLA-DQ	1.21	0.46–3.21	0.7012
	DQA1*05/DQB1*03:01 (DQ7) MM	1.47	0.65–3.29	0.3527
	DQA1*05/DQB1*03:01 (DQ7)/DQB1*02 (DQ2)	0.94	0.43–2.05	0.8686
	DQA1*05/DQB1*03 (DQ3)/DQB1*02 (DQ2) MM	0.90	0.41–1.96	0.7887
Graft loss (CLAD or death)				
	Age	1.33	0.94–1.88	0.1021
	Sex	0.65	0.34–1.25	0.1991
	CMV	1.07	0.46–2.46	0.8777
	HLAMatchmaker total score	1.05	0.68–1.61	0.8389
	HLAMatchmaker DQB1 score	0.99	0.62–1.56	0.9499
	HLA-EMMA total score	1.18	0.75–1.84	0.4768
	HLA-EMMA DQB1 score	1.16	0.68–1.97	0.5915
	PIRCHE-II total score	0.98	0.61–1.59	0.9457
	PIRCHE-II DQB1 score	1.12	0.46–2.72	0.6284
	KIR ligand HvG mismatch 1 MM 2 MM	1.18 2.13	0.61–2.26 1.00–4.54	0.6284 0.0496
	DSA anti-HLA-DQ			0.7975
	DQA1*05/DQB1*03:01 (DQ7) MM	1.36	0.66–2.78	0.4031
	DQA1*05/DQB1*03:01 (DQ7)/DQB1*02 (DQ2)	0.94	0.48–1.86	0.8601
	DQA1*05/DQB1*03 (DQ3)/DQB1*02 (DQ2) MM	0.90	0.46–1.78	0.7669
Time to first anti-HLA-DQ DSA				
	Age	0.91	0.64–1.31	0.6434
	Sex	1.11	0.45–2.69	0.8255
	CMV	1.03	0.34–3.10	0.9594
	HLAMatchmaker DQB1 score	1.44	0.77–2.67	0.2534
	HLA-EMMA DQB1 score	2.34	1.13–4.84	0.0215
	PIRCHE-II DQB1 score	2.17	1.11–4.24	0.0233
	KIR ligand HvG mismatch 1 MM 2 MM	0.43 0.00	0.17–1.09 1.88*10^−^20–2.47*10^13^	0.0767 0.7078
	DSA anti-HLA-DQ	4.37*10^5^	2.46*10^−^27–7.76*10^37^	0.7317
	DQA1*05/DQB1*03:01 (DQ7) MM	2.31	0.92–5.78	0.0737
	DQA1*05/DQB1*03:01 (DQ7)/DQB1*02 (DQ2)	1.38	0.56–3.40	0.4823
	DQA1*05/DQB1*03 (DQ3)/DQB1*02 (DQ2) MM	1.32	0.54–3.25	0.5436
Time to first biopsy-proven acute rejection				
	Age	0.90	0.67–1.19	0.4566
	Sex	1.23	0.57–2.67	0.5941
	CMV	1.05	0.39–2.79	0.9260
	HLAMatchmaker total score	1.45	0.88–2.39	0.1413
	HLAMatchmaker DQB1 score	0.88	0.50–1.53	0.6515
	HLA-EMMA total score	1.08	0.63–1.84	0.7835
	HLA-EMMA DQB1 score	0.84	0.44–1.58	0.5879
	PIRCHE-II total score	1.17	0.67–2.05	0.5570
	PIRCHE-II DQB1 score	0.90	0.49–1.64	0.7320
	KIR ligand HvG 1 MM 2 MM	1.18 2.53	0.54–2.57 1.05–6.08	0.6717 0.0383
	DSA DQ	0.86	0.30–2.51	0.7839
	DQA1*05/DQB1*03:01 (DQ7) MM	1.34	0.58–3.09	0.4926
	DQA1*05/DQB1*03:01 (DQ7)/DQB1*02 (DQ2)	1.19	0.55–2.61	0.6561
	DQA1*05/DQB1*03 (DQ3)/DQB1*02 (DQ2) MM	1.35	0.62–2.93	0.4450

Legend: Adjusted Cox proportional hazards models (adjusted for covariates sex, age, HLA sensitization and CMV status) regarding the outcomes of interest. CI, confidence interval; CLAD, chronic lung allograft dysfunction; DSA, donor-specific anti-HLA antibodies; HLA, human leukocyte antigen; HR, hazard ratio; HvG, Host-versus-Graft; KIR, Killer-cell immunoglobulin-like receptors, MM, mismatch; PIRCHE-II, Predicted Indirectly ReCognizable HLA epitopes presented by HLA class II molecules.

**FIGURE 1:**
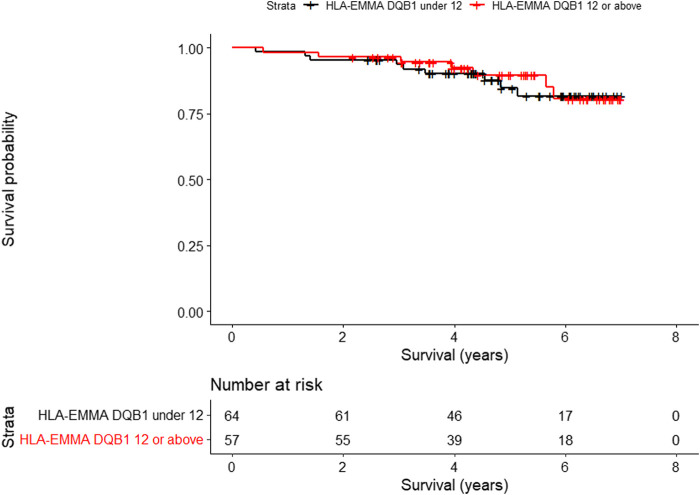
Kaplan-Meier analysis of HLA-EMMA DQB1 to overall survival (p = 0.0273) using the median of 12 as cutoff.

For overall survival, only HLA-EMMA DQB1 score (HR, 2.49; 95% CI, 1.11–5.59; P, 0.0273), was significantly associated with worse survival. Figure 1 shows the Kaplan-Meier analysis of HLA-EMMA DQB1 to overall survival using the median of 12 as cutoff. For CLAD, no association was seen between HLA compatibility scores and freedom from CLAD. For graft survival, only KIR ligand HvG when 2 mismatches were present (HR, 2.13; 95% CI, 1.00–4.54): P, 0.0496) was significantly associated with CLAD or death.

### Association of HLA Compatibility Scores With Time to *De Novo* DSA and Biopsy-Proven Acute Rejection

For the 120 patients in whom no DSA were detected pre-transplant, post-transplant anti-HLA antibody data were available (i.e. 1 patient had no post-transplant HLA data available). Of these, there were 24 patients (20%) in whom post-transplant DSA were detected. Three patients (13%) developed only HLA class I DSA, 1 patient (4%) developed only anti-HLA-DR DSA, and 20 patients (83%) developed anti-HLA-DQ DSA. Only 5 of the 20 patients (25%) with anti-HLA-DQ DSA developed CLAD by the end of the study and 1 patient (5%) deceased. However, we observed that these antibodies are mostly undetectable over time. Three of the 5 patients with HLA-DQ antibodies who developed CLAD (60%) had anti-HLA-DQ antibodies that were permanently detectable with an MFI value >7000 once in the follow-up period.

For time to dnDSA, HLA-EMMA DQB1 score and PIRCHE-II DQB1 score were associated with more rapid development of anti-HLA-DQ antibodies (HLA-EMMA DQB1 scores HR, 2.34; 95% CI, 1.13–4.84; P, 0.0215) (PIRCHE-II DQB1 scores HR, 2.17; 95% CI, 1.11–4.24; P, 0.0233). Regarding the specific HLA-DQ mismatches, we noticed a higher association with HLA-DQA1*05/DQ7 mismatch (HR, 2.31; 95% CI, 0.92–5.78; P, 0.0737) than with DQA1*05/DQ7/DQ2 (HR, 0.94; 95% CI 0.43–2.05; P, 0.8686) and DQA1*05/DQ3/DQ2 (HR, 0.90; 95% CI, 0.41–1.96; P, 0.7887) mismatches.

For time to first biopsy-proven rejection episode, only KIR ligand HvG when 2 mismatches were present (HR, 2.53; 95% CI, 1.05–6.08): P, 0.0383) was significantly associated with either cellular/ACR or antibody-mediated/AMR. Among which, 8 patients showed AMR (definite, n = 0; probable, n = 4; possible, n = 4), and 24 patients showed ACR (A0B1, n = 5; A0B2, n = 1; A0B3, n = 1; A1B0, n = 8; A1B1, n = 1; A1B2, n = 1; A1Bx, n = 1; A2B0, n = 3; A2Bx, n = 1; A3B1, n = 1; AxB2, n = 1).

## Discussion

In this single-center lung transplant cohort we demonstrated that HLA-EMMA DQB1 score was significantly associated with worse survival and more rapidly developing anti-HLA-DQ antibodies after lung transplantation. Also, the PIRCHE-II DQB1 score was significantly associated with time to *de novo* anti-HLA-DQ DSA. Although other results with B- and T-cell epitope mismatch scores were not significant, we observed higher hazard ratios regarding overall survival and time to *de novo* anti-HLA-DQ DSA when scores were calculated considering only the HLA-DQB1 locus. This is in line with the finding that 83% of included patients developing dnDSA presented with anti-HLA-DQ DSA.

A potential rationale why HLA-EMMA DQB1 score gave a significant result and not HLAMatchmaker DQB1, two different software tools for calculating the HLA B-cell epitope mismatch score, is that HLAMatchmaker postulates that eplets as defined by the HLA Eplet Registry^4^ have immunogenic significance and are distinct from the ‘structural epitope’ which refers to the full footprint of the area recognized by an antibody [23, 24]. HLA-EMMA, on the contrary, does the calculation at the solvent accessible amino acid level, so potential bias of these eplets is excluded [16].

Previous research has demonstrated that not all molecular mismatches equally contribute to the generation of donor-specific immune responses and that immunogenicity is not merely a quantitative issue, but that one or only a few epitope mismatches are sufficient to induce an antibody response. We therefore also looked specifically at the mismatches considered in the literature as so-called high-risk epitope mismatches (REMs) [20–22, 25]. For overall survival, CLAD, graft survival and time to biopsy-proven acute rejection, no significant associations with REMs were found. For time to *de novo* anti-DQ-HLA DSA, we observed a trend for an association with HLA-DQA1*05/DQ7 mismatch (HR, 2.3; 95% CI, 0.92–5.78; P, 0.0737), more than with DQA1*05/DQ7/DQ2 (HR, 0.94; 95% CI 0.43–2.05; P, 0.8686) and DQA1*05/DQ3/DQ2 (HR, 0.90; 95% CI, 0.41–1.96; P, 0.7887) mismatches.

Our results partly align with similar observations in the kidney/lung transplant literature, identifying HLA-DQ mismatches and HLA-DQ mismatch load as risk factors for dnDSA development and poor allograft outcome [20–22]. The study on lung transplant recipients from Hiho et al. [26] showed that a lower number of HLA class II mismatches (specifically HLA-DR and -DQ) for all approaches (HLAMatchmaker, HLA-EMMA, PIRCHE-II) was associated with a reduced risk of restrictive allograft syndrome (restrictive phenotype of CLAD), DSA development, and improved overall survival. The lung transplant studies from Bedford et al. [27], Kleid et al [28]. and Lobashevsky et al. [29] showed an association between a higher epitope mismatch load and an increased risk of dnDSA development. These results were more pronounced with HLA class II [28] and HLA-DQ (HLA-DQA1*05 + HLA-DQB1*02/03:01) mismatches [27]. Further studies with larger cohorts are needed to further unravel the importance of these HLA-DQ compatibility scores and specific HLA-DQ mismatches.

A limitation of our study, which may affect the strength of our observations and may explain why some of the reported statistical differences are marginal, is the limited number of included patients (n = 128) which may hinder the analysis of subtle outcome differences (low event numbers for some endpoints) in multi-confounding endpoints like graft survival. Lack of inclusion of other competing risk factors (levels of immunosuppression, competing immune events such as infection, etc.), and HLA expression of HLA molecules on the donor lung influenced by the degree of inflammation and T-cell activation upon transplantation [30], may influence the observed transplant outcome and may hinder analysis of HLA compatibility. DSA may also not be detected because of phasic release and DSA adsorption/precipitation in the graft due to the ‘sponge effect’ related to the higher capillary surface in the lung [31, 32] or the DSA may be antibodies to self-antigens or non-HLA antigens, which can also lead to CLAD after lung transplantation [33–35].

Regarding missing self-induced rejection by NK cells (KIR ligand Host-versus-Graft mismatch), we saw only a significant association for graft survival (CLAD or death) and for time to first biopsy-proven rejection episode when 2 mismatches were present. We also observed a higher hazard ratio for overall survival (HR, 2.79; 95% CI, 0.95–8.17; P, 0.0616) and CLAD (HR, 2.16; 95% CI, 0.91–5.10; P, 0.0799) when 2 mismatches were present. In addition to the limitations described above, insufficient priming events and insufficient number of NK cells may affect our results. Recent experimental evidence has demonstrated that educated NK cells need to undergo priming such as ischaemia/reperfusion injuries and viral infections to acquire their full effector functions, in addition to individual heterogeneity of the NK cell population [4]. In contrast to previous research in kidney transplantation [4, 36], we did not perform any KIR gene sequencing and expression testing, which would be necessary for accurate determination of mismatch scores. The KIR ligand calculation we used was based on KIR ligands grouped into 3 major categories based on the KIR-binding epitope in HLA-C and HLA-B [17–19]. The impact of missing self-induced rejection by NK cells warrants further investigation.

In summary, despite the limitations related to its retrospective design, our study suggests that HLA-DQB1 compatibility scores and KIR ligand HvG 2 mismatches at the time of transplant may allow for identifying recipients at risk of poor long-term outcomes after lung transplantation. These data indicate that HLA-DQB1 compatibility scores and KIR ligand HvG two mismatches could become useful for risk stratification after lung transplantation, which could potentially translate into the recommendation of close surveillance and/or fine-tuning of immunosuppressive regimens in this immunologically high-risk population to improve survival, but further validation in independent cohorts is necessary.

## Data Availability

The raw data supporting the conclusions of this article will be made available by the authors, without undue reservation.

## Footnotes

1
http://www.epitopes.net

2
https://hla-emma.com/

3
https://www.pirche.com

4
https://epregistry.com.br

## Abbreviations

CLAD, Chronic lung allograft dysfunction; HLA, Human leukocyte antigen; DSA, Donor-specific anti-HLA antibodies; PIRCHE-II, Predicted Indirectly ReCognizable HLA epitopes presented by HLA class II molecules; dn, *De novo*; ADCC, Antibody-dependent cell-mediated cytotoxicity; CDC, Complement-dependent cytotoxicity; AMR, Antibody-mediated rejection; ACR, Acute cellular rejection; NK cell, Natural killer cell; KIRs, Killer-cell immunoglobulin-like receptors; EFI, European Federation for Immunogenetics; CMV, Cytomegalovirus; HR, Hazard ratio; CI, Confidence interval; REM, Risk epitope mismatch; MM, Mismatch; HvG, Host-versus-Graft; MFI, Median Fluorescence Intensity; ISHLT, International Society for Heart and Lung Transplantation.
